# Socio-demographic and lifestyle factors for child’s physical growth and adiposity rebound of Japanese children: a longitudinal study of the 21st century longitudinal survey in newborns

**DOI:** 10.1186/1471-2458-14-334

**Published:** 2014-04-09

**Authors:** Yoko Franchetti, Hiroo Ide

**Affiliations:** 1 Department of Biostatistics & Computational Biology, Dana-Faber Cancer Institute, 450 Brookline Ave., Boston, MA, USA; 2 Department of Biostatistics, Harvard School of Public Health, 677 Huntington Ave., Boston, MA, USA; 3 Research Division of Health Policy in Aging Society, Chiba University Hospital, Inohana 1-8-1, Chuo-ku, Chiba-shi, Chiba, Japan

**Keywords:** Obesity, Adiposity rebound (AR), Body mass index (BMI), Longitudinal survey, Lifestyle

## Abstract

**Background:**

It is unknown whether childhood physical development in Asian populations differs from western populations, since no longitudinal analysis has been performed in Asian countries yet. Utilizing *the 21st Century Longitudinal Survey in Newborns*, we studied the timing of adiposity rebound (AR) among Japanese children and determined whether AR occurs earlier in obese children compared to nonobese children. Furthermore, we identified important demographic, social, and lifestyle factors that affect their physical development.

**Methods:**

We used data from the annual surveillance of Japanese children born in 2001, with 45,392 eligible subjects. We applied survival analysis to evaluate the AR and a trajectory method for the BMI transition across 5 ½ years. Time-dependent and time-independent factors affecting BMI changes were investigated using longitudinal analysis. Accounting for the known difference in prevalence between Japanese and Western children, we adopted a 95^th^ percentile of BMI as criterion for obesity.

**Results:**

Mean BMI at birth and at ages 1 ½, 2 ½, 3 ½, 4 ½, and 5 ½ years for all subjects were 12.6, 16.3, 16.1, 15.8, 15.5, and 15.4, respectively, showing a progressive reduction after 1 ½ years. However, among obese children at 5 ½ years, 39.6% had experienced AR as early as at age 4 ½ years. Controlling for sex, Cox’s proportional hazards model showed that obese children had a 48.5% higher hazard to experience AR than nonobese children. The difference in BMI transition between obese and non-obese children was also captured by a trajectory method. In longitudinal analysis, BMI was lower for children who had a longer gestational period whereas children who received parental care from non-family members gained higher BMI values.

**Conclusions:**

With the 95^th^ percentile cutoff for children obesity, obese Japanese children developed AR earlier than nonobese Japanese children, similar to those in Western countries reported in the literature. Primary caretakers and length of gestational period were the most important socio-demographic factors affecting physical development.

## Background

Obesity is a substantial risk factor for lifestyle disease and it predicts the development of obesity in adulthood [1,2]. Childhood obesity is also associated with metabolic syndrome including hyperinsulinemia/insulin resistance, dyslipidemia, and hypertension. Thus, risks related to lifestyle diseases are considered to be present from childhood [3,4]. Adiposity rebound (AR) is the phenomenon in which the body mass index (BMI) increases after reaching its minimum level during childhood. Several studies have suggested that the risk of obesity in Western countries be predicted by observing AR [5]. On the other hand, to the best of our knowledge no systematic, *longitudinal* study has been conducted in Asian populations to conclude that early AR is a significant factor to differentiate for childhood obesity.

For Western populations, for instance, Rolland-Cachera et al. found that children who experienced AR earlier had a higher adiposity at age 16 years compared to those who experienced a later AR [5]. Prokopec and Bellisle showed that there was an inverse relationship between age at AR and BMI in adulthood while BMI itself varied during growth in an European population [2]. Accordingly, AR may be a useful indicator associated with lifestyle diseases through high adult BMI [6-8]. A recent review by Brisbois et al. concluded that AR, maternal BMI, and father’s employment were associated with adult obesity [9]. Among these factors, only AR is a practical candidate indicator that can be used for intervention in children to prevent adult obesity. Children who experience AR earlier may represent a high-risk group, and measures to prevent obesity may be implemented for these individuals.

Distribution of BMI in children and timing of AR are known to vary across regions and ethnic backgrounds [10]. According to the recent cross-sectional analysis by Pan et al. [11], BMI increases at a higher rate in Chinese children than in those in Western countries. Another study showed the prevalence of obesity in Japanese children has clearly increased and it differs from in the United States, Brazil, and China [12]. While cross-sectional analyses were used in these studies, it is essential to elucidate a causal relationship between AR and BMI change using a longitudinal study on demographic, social, and lifestyle factors so that intervention targets and preventive measures against obesity be established [13]. In a well-known epidemiological research project called the Avon Longitudinal Study of Pregnancy and Childhood (ALSPAC), some socio-demographic and lifestyle factors have been studied: using a subset of the cohort, an European study team analyzed diet and socio-economic statues as candidate factors for affecting BMI change and concluded that these factors were not associated with BMI change [14]. Another ALSPAC study team identified early life environment as a predictor of late obesity, although in a cross-sectional fashion, and commented that the list of potential risk factors and intervention targets should be extended [15].

In this study, our goal was to longitudinally study whether AR occurs earlier in obese children compared to nonobese children in the Japanese population. Furthermore, we aim to identify demographic, social, and lifestyle factors that affect the growth of their BMI. To compensate for ethnic differences we used a BMI cutoff different from the IOTF and WHO standards but based on the literature for this population [16] so that the difference in children BMI between Western and Japanese populations can be accounted for. We used data from *the 21st Century Longitudinal Survey in Newborns* collected and maintained by the Japanese government (Household Statistics Office, Vital, Health and Social Statistics Division, Statistics and Information Department, Ministry of Health, Labour and Welfare, Japan). This is the first longitudinal nationwide public survey for understanding lifestyle and environment of children and designing appropriate family policies in Japan. In this paper, we analyzed the data from 2001 to 2006, applying longitudinal analytical methods.

## Methods

### Subjects

The 21st Century Longitudinal Survey in Newborns started with Japanese newborns born in 2001; their physical, socioeconomic, and lifestyle data were collected and followed annually by the Japanese government. All children born in Japan between January 10 and January 17 and between July 10 and July 17, 2001 were enrolled. Information was obtained from postal questionnaires. The survey will continue until the subjects turn 20 years old. Our research used the data obtained from 2001 to 2006, i.e., collected at ages 6 months, and 1 ½, 2 ½, 3 ½, 4 ½, and 5 ½ years on average. A total of 53575 subjects were included in the baseline survey, 47015 (87.8%) responded to the questionnaires, and 99.6 percent of respondents were their parents. Among the responders at baseline, 43925 (93.9%), 42812 (91.1%), 41559 (88.4%), 39817 (84.7%), 38537 (81.2%) responded to the follow-up questionnaires at ages 1 ½, 2 ½, 3 ½, 4 ½, and 5 ½ years, respectively. The survey contained information on parents’ nationality, and not their ethnicity. However, 98.5% of Japanese citizens are ethnical Japanese [17]. This study used the data of 45392 children (23608 boys, 21784 girls) whose parents were Japanese, and their birth height, weight, and gestational period were recorded in the survey of age 6 months.

All respondents consented to the purpose of the 21st Century Longitudinal Survey in Newborns described by the Japanese government. Our research used the data of the 21 Century Longitudinal Survey in Newborns, which was conducted by the Japanese government and completely anonymized and de-identified when provided by the government to the authors. Direct access to the database on the survey is not permitted to any third party; the dataset for our study was provided by Ministry of Health, Labour and Welfare through our Health and Labour Sciences Research Grant from 2008 to 2011. The authors complied with the Japanese Personal Information Law to take additional measures to protect individual privacy. No ethical approval or consent was required for the analysis in this manuscript according to the Ethical Guidelines for Epidemiological Studies in Japan (issued by the Ministry of Health, Labour, and Welfare and the Ministry of Education, Culture, Sports, Science and Technology, Japan, issue date: June 17, 2002, the fourth amendment date: December 1, 2008) [18].

### Data management

BMI values were calculated from the parental reported (self-reported) height and weight at birth (all subjects were born in the month of July or January in 2001) and at 1 ½, 2 ½, 3 ½, 4 ½ and 5 ½ years of age based on the following formula:

$$\text{BMI}=\text{weight}\;\left(\text{kg}\right)/\text{height}\;{\left(m\right)}^{2}$$

In addition to the above characteristics, we chose candidate variables for explaining physical development from the 21st Century Longitudinal Survey in Newborns. The survey collected parents’ income at ages 6 months and 4 ½ years. Parents’ income was classified into five groups based on Japanese yearly family income quintiles [19]. The thresholds on parents’ income were 4.61, 5.87, 6.97, and 8.65 million yen (about 42000, 53000, 63000, and 79000 USD, respectively) at age 6 months and 4.66, 5.77, 6.81, and 8.38 million yen (about 42000, 52000, 62000, and 76000 dollars, respectively) at age 4 ½ years. Threshold incomes were converted to USD by purchasing power parity rate (1 USD = 110 yen). Data on monthly expenses for raising children were collected from the 1st through 6th follow-up surveys and were used as a *time-dependent* explanatory variable in longitudinal analysis.

We created summary categories based on the original survey questions. A child’s main parental care provider during weekdays from the 1st to 6th follow-up surveys was classified into father, mother, grandfather, grandmother, or other; sibling was classified into present or absent; family was classified on the basis of three-generation family, nuclear family, single parent, or other. These measurements were also used as time-dependent explanatory variables in longitudinal analysis.

Some questionnaire items appeared only at 5th follow-up surveys and were used as *time-invariant* explanatory variables in longitudinal analysis. The 5th follow-up survey-specific variables were wake-up time, bedtime, time spent watching television, time spent playing video games, and consumption of breakfast, lunch, dinner, and snacks; wake-up time was classified into before 7, 7–8, 8–9, after 9 a.m., or irregular for descriptive statistics and converted to continuous hours for longitudinal modeling; time spent watching television was originally classified into 0, <1, 1–2, 2–3, 3–4, 4–5, or ≥5 hours; that playing video games was classified into 0, <1, 1–2, 2–3, or ≥3 hours. Consumption of breakfast, lunch, dinner, and snacks was classified into regular, irregular, or no consumption. Sleeping hours were calculated using wake-up time and bedtime reported at the same follow-up survey.

### Statistical analysis

Cross-sectional distribution of BMI was evaluated as follows. We obtained the 25^th^, median, 75^th^, 85^th^, 95^th^, and 99^th^ percentile of BMI for both sexes. Since the prevalence of obesity in Japanese children is reported to be different from other countries [12], we classified the subjects into two groups such as ≥85^th^ percentile (overweight) versus < 85^th^ percentile and ≥95^th^ percentile (obese) versus < 95^th^ percentile at age 5 ½ years [16], instead of using the IOTF and WHO standards [10,20]. We provide descriptive statistics on obesity and overweight using the IOTF and WHO standards in Table 1 only as a reference to compare with other studies.

**Table 1:** Distribution of body mass index among children participating in the current study

	WHO standard*									
	**Boy (%)**					**Girl (%)**				
	**1 ½ years**	**2 ½ years**	**3 ½ years**	**4 ½ years**	**5 ½ years**	**1 ½ years**	**2 ½ years**	**3 ½ years**	**4 ½ years**	**5 ½ years**
99 percentile-	1.9	2.6	2.0	1.8	2.4	1.6	1.8	1.3	1.1	1.3
95 percentile -	4.8	5.9	4.9	3.7	3.2	4.7	5.6	3.9	3.1	2.5
85 percentile -	12.9	13.6	12.5	9.3	8.5	11.7	14.3	11.6	8.1	7.4
75 percentile -	12.0	11.2	12.6	11.3	9.5	10.9	12.3	11.5	11.0	9.5
50 percentile -	26.2	28.7	28.0	29.0	27.2	28.9	24.9	31.7	28.9	27.9
25 percentile -	22.6	19.9	22.9	28.2	26.8	25.2	24.1	22.7	29.9	29.0
< 25 percentile	19.5	18.2	17.1	16.8	22.4	17.1	17.0	17.3	18.0	22.5
	**IOTF standard**^**†**^									
	**Boy (%)**				**Girl (%)**					
	**2 ½ years**	**3 ½ years**	**4 ½ years**	**5 ½ years**	**2 ½ years**	**3 ½ years**	**4 ½ years**	**5 ½ years**		
Obesity	1.3	1.0	1.2	1.6	1.2	1.1	1.3	1.6		
Overweight	7.2	5.9	5.0	5.1	8.5	7.2	7.6	7.5		

*The WHO defines two different standards for younger children (0–5 years) and older children and adolescent (5 years -). We applied “WHO growth reference for school-aged children and adolescents” for the Japanese children age at 5 ½ years, and “WHO child growth standards based on length/height, weight and age” for those at the rest of the ages [19,21].

†The IOTF standard provides equivalent BMI cutoff values, 25 kg/m^2^ for overweight and 30 kg/m^2^ for obesity, at the age of 18 years for both boys and girls [10].

AR was defined as the event where annually reported BMI shows a positive change after reaching its minimum level from baseline BMI. We determined the individual timings of AR, created a binary event variable (AR event versus censored), and performed survival analysis using Kaplan-Meier method with log-rank tests for significance between groups. For estimating hazard ratios, Cox’s proportional hazard models were also used. In order to capture BMI trends, we used a trajectory model which is based on a discrete mixture model and has been used to model long-term human activities such as offensiveness and growth in children [22-24]. Trends in BMI growth were evaluated for both sexes.

To model longitudinal data, we used generalized estimating equations (GEEs). We chose GEE models since the models provide robust parameter estimates regardless of our assumed variance-covariance correlation matrix whereas other commonly used longitudinal models, random effects models, may be sensitive to a particular choice of the correlation structure [25,26]. Furthermore, our large nation-wide data with the high response rate fits the assumption of missing completely at random (MCAR), a required assumption for using GEE models. Note that MCAR generally does not produce biased estimates of mean effects. Simply using observed samples is valid for data with MCAR, and a sensitivity analysis with missing data analysis is not necessary [27,28]. The assumption of MCAR was tested by the proportions of missing in BMI over time using a *χ*^2^-test [29]. It was also tested by the proportions of missing in BMI at baseline across five family income strata that were available at the closest follow-up time point using a *χ*^2^-test [30]. Based on non-significant results of these statistical tests (*P* =1.00 and *P* = .39, respectively), it was plausible to assume that our data was MCAR and that socio-economic status did not affect missing data mechanism in our outcome.

Using GEE models, we investigated factors that affected BMI. Attribution (gestational period, baseline and concurrent parental care provider, sibling, and household member), economic situation (baseline and the 5th year parents’ income, baseline and concurrent expenses for a child), and lifestyle (television watching, video game, wake-up time, sleeping hours, and consumption of breakfast, lunch, dinner, and snacks at the 5th follow-up survey) were considered as potential explanatory variables whereas concurrent BMI from ages 1 ½ years through 5 ½ years was treated as a dependent variable. The GEE models were further adjusted for BMI at birth, concurrent age, overweight/not overweight at age 5 ½ years, and sex. In all GEE models the exchangeable variance-covariance structure was specified due to the stability in convergence. There is no step-wise program for GEE models, so we manually performed step-wise covariate selection based on p-values. The final GEE model was chosen by retaining significant candidate explanatory variables from the fit longitudinal models. All data were analyzed by SAS 8.2 (SAS Institute Inc., USA) and STATA 11.0 (StataCorp LP., USA) software. The level of statistical significance was set at *P* < .05.

## Results

Table 2 gives descriptive statistics of BMI for all children, BMI stratified by gender, parents’ annual income, sleep hour, expense for child, gestational period, birth weight, main parental care provider, sibling, household member, wake-up time, television watching, video game playing, breakfast, lunch, dinner, and snack. Children with birth weight >2500 g accounted for 91.5% of the sample. Mean BMI of all subjects from birth to age 5 ½ years showed a progressive reduction after age 1 ½ years from 16.33 kg/m^2^ to 15.38 kg/m^2^. The proportion of main parental care provider changed from mother (92.2%) at the 1st follow-up survey to mother (40.9%) and others (56.3%) at the 5th follow-up survey. There was no significant difference in household member between the 1st and 5th follow-up surveys. Time spent watching television was merely correlated with the time spent playing video games (correlation coefficient, 0.124; *P* < .01).

**Table 2:** Descriptive statistics of children and their households participating in the current study

		**All**			**Boys**			**Girls**		
**Variable**	**Age at survey**^**†**^	**N**	**Mean**	**SD**	**N**	**Mean**	**SD**	**N**	**Mean**	**SD**
Body mass index (kg/m^2^)	At birth	45284	12.62	1.23	23542	12.65*	1.23	21742	12.58*	1.24
	1 ½ years	38422	16.33	1.45	19939	16.52*	1.43	18483	16.13*	1.44
	2 ½ years	33672	16.09	1.45	17523	16.21*	1.43	16149	15.96*	1.45
	3 ½ years	36146	15.75	1.33	18797	15.82*	1.32	17349	15.66*	1.33
	4 ½ years	35097	15.48	1.36	18265	15.52*	1.34	16832	15.44*	1.37
	5 ½ years	33057	15.38	1.50	17212	15.43*	1.50	15845	15.34*	1.50
**Variable**	**Age at survey**^**†**^	**Unit/category**	**N**	**Mean (SD) or %**						
Parents’ annual income	½ year	(million yen)	45383	5.30 (3.28)						
	4 ½ years		36922	5.77 (3.47)						
Sleep hour										
	4 ½ years	(hours)	36026	9.9 (0.8)						
Expense for child										
	½ year	(10,000 yen/month)	44093	2^‡^ (0, 400)^‡^						
	4 ½ years		37347	4^‡^ (0, 447)^‡^						
Gestational period	--	22-36 weeks	45392	5.1						
		37-41 weeks		94.1						
		≥ 42 weeks		0.9						
Birth weight	--	< 1000 g	45392	0.2						
		1001-1500 g		0.4						
		1501-2500 g		7.9						
		≥ 2501 g		91.5						
Main parental care provider	½ year	Mother	45316	92.2						
		Father		0.1						
		Grandmother		3.7						
		Grandfather		0.1						
		Others		3.9						
	4 ½ years	Mother	38296	40.9						
		Father		0.3						
		Grandmother		2.4						
		Grandfather		0.2						
		Others		56.3						
Sibling	½ year	No sibling	45392	49.6						
		With sibling		50.4						
	4 ½ years	No sibling	38715	18.7						
		With sibling		81.3						
Household member	½ year	With grandparent (s) and parent(s)	45392	21.6						
		With both parents		77.9						
		With a parent		0.4						
		Other		0.1						
	4 ½ years	With grandparent(s) and parent(s)	38715	23.0						
		With both parents		73.9						
		With a parent		3.0						
		Other		0.2						
Wake-up time	4 ½ years	Before 7 am	38634	25.1						
		7 am-		60.7						
		8 am-		11.0						
		9 am-		1.0						
		Irregular		2.2						
Television watching^§^	4 ½ years	None	37377	1.1						
		< 1 hour		15.9						
		1 hour		40.2						
		2 hours		28.1						
		3 hours		10.5						
		4 hours		3.2						
		≥ 5 hours		1.0						
Video game playing	4 ½ years	None	37240	60.5						
		< 1 hour		29.7						
		1 hour		8.5						
		2 hours		1.1						
		3 hours		0.3						
Breakfast	4 ½ years	Eating regularly	45392	97.7						
		Eating irregularly		1.6						
		Don’t eat		0.7						
Lunch	4 ½ years	Eating regularly	45392	99.4						
		Eating irregularly		0.6						
		Don’t eat		0.0						
Dinner	4 ½ years	Eating regularly	45392	97.4						
		Eating irregularly		2.6						
		Don’t eat		0.1						
Snack	4 ½ years	Eating regularly	45392	90.8						
		Eating irregularly		7.9						
		Don’t eat		1.3						

*Statistically significant at the level of *P* < .05 in a two-sided *t*-test between genders.

^†^Survey time points were at approximate mean ages.

^‡^We described the median, the maximum, and the minimum values because the data did not follow a normal distribution.

^§^Spearman’s correlation with time spent playing video games was 0.124 (*P* < .01).

**Figure 1:**
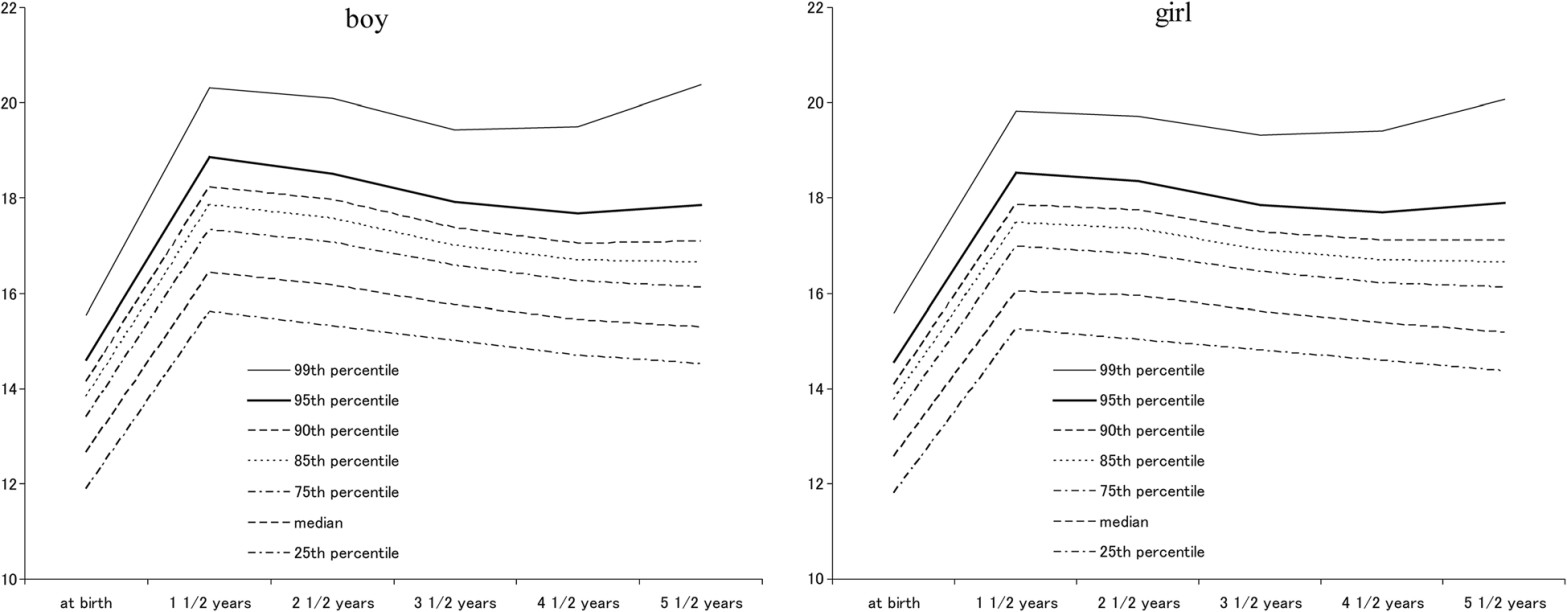
Trends in cross-sectional BMI by sex from baseline to the 6th follow-up survey.

As shown in Figure 1, the 99^th^ percentile of BMI was the lowest at age 3 ½ years, and the 90-95^th^ percentiles were the lowest at age 4 ½ years and increased thereafter. BMI values were significantly higher in boys than girls in all follow-up surveys (*P* < .01). In children in the 85-95^th^ percentiles of BMI, BMI was the lowest between ages 4–6 years. No AR was observed during 0–6 years in nonoverweight boys and girls in the cross-sectional distribution.

**Figure 2:**
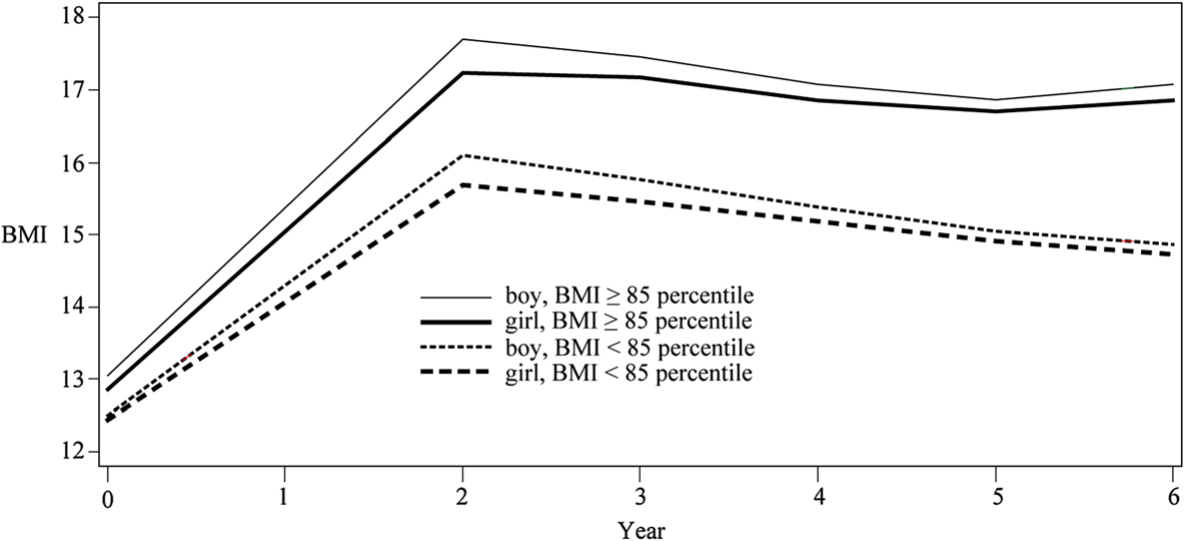
Trajectory models of BMI for overweight children and nonoverweight children by sex during six-year surveys.

In survival analysis, time to AR significantly differed between obese and nonobese children (*P* < .01); at age 4 ½ years 39.6% of obese children had experienced AR while 30.8% of nonobese children had experienced AR as in Table 3. Controlling for sex, Cox’s proportional hazards model showed that obese children had a 48.5% higher hazard to experience AR than nonobese children (hazard ratio, 1.485; 95% confidence interval, 1.267 - 1.335). When included in the same model, interaction between obesity and sex was not significant. The results of Cox’s proportional hazards models controlling for sex (without interaction terms) are summarized in Table 4. The trajectory analysis for obese children demonstrated a typical growth pattern of AR, with an increase, a decrease, then a second increase in BMI. The same growth pattern was observed for overweight children. No difference was observed between sexes as in Figure 2.

**Table 3:** Cumulative prevalence rate of adiposity rebound (AR) stratified by overweight and by obesity

**Mean age**	**Overweight (N = 4,701)**	**Non overweight (N = 26,570)**
2 ½ years	0.0%	0.1%
3 ½ years	23.8%	21.4%
4 ½ years	35.8%	30.4%
**Mean age**	**Obesity (N = 1,523)**	**Non obesity (N = 29,748)**
2 ½ years	0.1%	0.1%
3 ½ years	25.7%	21.6%
4 ½ years	39.6%	30.8%

**Table 4:** Hazard ratios of adiposity rebound (AR) by overweight and by obesity controlling for sex

**Cox’s proportional hazard models**^**†**^	**Comparison**	**Hazard ratio**
		**(95% confidence interval)**
Model 1	Overweight versus nonoverweight	1.300 (1.267 - 1.335)*
	Girls versus boys	1.099 (1.077 - 1.122)*
Model 2	Obesity versus non obesity	1.485 (1.426 - 1.547)*
	Girls versus boys	1.098 (1.076 - 1.121)*

*Statistically significant at the level of *P* < .05.

†No interaction term is included.

N = 31,271.

In our final longitudinal model, the results of GEE analysis demonstrated that gestational period was negatively correlated with BMI; 0.160 for <22-36 weeks (*P* < .01) and -0.144 for ≥42 weeks (*P* < .01) comparing to 37–41 weeks, suggesting that BMI was smaller for children with a longer gestational period. Of parental care provider, categories of nonfamily member at age 6 months (0.122; *P* < .01) and during the entire study period (0.087; *P* < .01) were significant. A category of 1–2 hours for time spent playing video games at age 4 ½ years was significant (0.068; *P* < .01). Wake-up time (-0.044; *P* < .01) was a significant predictor of BMI; children who awoke 1 hour later had a 0.04 lower BMI. Irregular snack consumption (-0.047; *P* = .01) was also a statistically significant factor. There were no significant associations between BMI and sleeping hours, time spent watching television, and regular diet (breakfast, lunch, and dinner). The results of GEE analysis are presented in Tables 5 and 6.

**Table 5:** The generalized estimating equation (GEE) of BMI for six-year surveys retaining significant exploratory factors

**Variable**	**Category**	**Estimate**	**95% confidence interval**		**P value**	**P value in type 3 effect analysis**^**‡**^
BMI^†^ at baseline survey		0.151	0.141	0.161	<.01*	<.01*
Age		-0.263	-0.268	-0.258	<.01*	<.01*
Overweight at age 5 ½ years		1.742	1.710	1.775	<.01*	<.01*
(Base category; < 85^th^ percentile)						
Gender (base category; boy)	Girl	-0.160	-0.181	-0.139	<.01*	<.01*
Gestational period	< 22–36 weeks	0.160	0.106	0.214	<.01*	<.01*
(Base category; < 37–41 weeks)	≥ 42 weeks	-0.144	-0.262	-0.003	<.01*	
Main parental care provider at age 6 months	Father	-0.107	-0.389	0.175	.46	<.01*
(Base category; mother)	Grandmother	0.024	-0.038	0.087	.44	
	Grandfather	0.013	-0.321	0.347	.94	
	Others	0.122	0.063	0.181	<.01*	
Main parental care provider across the surveys	Father	-0.039	-0.186	0.108	.60	<.01*
(Base category; mother)	Grandmother	-0.055	-0.100	-0.010	.02*	
	Grandfather	0.108	-0.053	0.268	.19	
	Others	0.087	0.071	0.102	<.01*	
Wake-up time (base time; 0 am)		-0.044	-0.062	-0.026	<.01*	<.01*
Video game playing	< 1 hour	0.014	-0.010	0.037	.25	.02*
	1 hour -	0.068	0.029	0.107	<.01*	
	2 hour -	0.009	-0.104	0.122	.88	
	3 hour -	-0.020	-0.265	0.225	.87	
Snack at age 4 ½ years	Eating irregularly	-0.047	-0.083	-0.010	.01*	.03*
(Base category; eating regularly)	Don’t eat	-0.044	-0.148	0.059	.40	

*Statistically significant at the level of *P* < .05.

^†^BMI: body mass index.

^‡^Type 3 effect test is based on an asymptotic chi-square distribution of the likelihood ratio statistic computed from GEE models and it allows one to test for *an effect of interest provided all the other variables are included in the GEE model*.

**Table 6:** The generalized estimating equation (GEE) of BMI for six-year surveys including all candidate exploratory factors

**Variable**	**Category**	**Estimate**	**95% confidence interval**		**P value**	**P value in type 3 effect analysis**^**§**^
BMI^†^ at baseline survey		0.150	0.139	0.161	<.01*	<.01*
Age		-0.263	-0.269	-0.258	<.01*	<.01*
Overweight at age 5 ½ years (base category; < 85^th^ percentile)		1.736	1.703	1.770	<.01*	<.01*
Gender (base category; boy)	Girl	-0.167	-0.189	-0.145	<.01*	<.01*
Gestational period	< 22–36 weeks	0.170	0.115	0.225	<.01*	<.01*
(Base category; 37–41 weeks)	≥ 42 weeks	-0.159	-0.278	-0.039	<.01*	<.01*
Main parental care provider at age 6 months	Father	-0.088	-0.383	0.208	.56	
(Base category; mother)	Grandmother	0.004	-0.061	0.069	.91	
	Grandfather	-0.012	-0.349	0.326	.95	
	Others	0.123	0.061	0.184	<.01*	
Main parental care provider across the surveys	Father	-0.094	-0.249	0.061	.24	<.01*
(Base category; mother)	Grandmother	-0.065	-0.112	-0.017	.01*	
	Grandfather	0.115	-0.052	0.283	.18	
	Others	0.084	0.067	0.100	<.01*	
Sibling at age 6 months (base category; no sibling)	With sibling	0.018	-0.008	0.043	.17	.17
Sibling across the surveys (base category; no sibling)	With sibling	-0.011	-0.032	0.011	.34	.34
Household member at age 6 months	With both parents	-0.026	-0.061	0.009	.15	.21
(base category; with grandparent(s) and parent(s))	With a parent	0.069	-0.107	0.244	.45	
	Other	-0.673	-1.285	-0.061	.03*	
Household member across the surveys	With both parents	-0.027	-0.058	0.004	.09	.33
(Base category; with grandparent(s) and parent(s))	With a parent	-0.040	-0.103	0.024	.22	
	Other	-0.079	-0.334	0.175	.54	
Parents’ income at age 6 months^‡^	4,610,000 yen-(42,000 dollars-)	-0.001	-0.033	0.030	.94	.79
(Base category; < 4,610,000 yen (about 42,000 dollars))	5,870,000 yen-(53,000 dollars -)	0.009	-0.027	0.046	.61	
	6,970,000 yen-(63,000 dollars -)	0.020	-0.019	0.058	.31	
	8,650,000 yen-(79,000 dollars -)	0.000	-0.047	0.046	.99	
Parents’ income at age 4 ½ years^‡^	4,660,000 yen-(42,000 dollars -)	0.022	-0.009	0.054	.16	.16
(Base category; < 4,660,000 yen (about 42,000 dollars))	5,770,000 yen-(52,000 dollars -)	0.033	-0.003	0.069	.07	
	6,810,000 yen-(62,000 dollars -)	0.007	-0.031	0.046	.71	
	8,380,000 yen-(76,000 dollars -)	0.045	0.000	0.090	.05	
Expense for kid at age 6 months		0.000	-0.001	0.002	.61	.61
Expense for kid across the surveys		0.000	-0.001	0.001	.56	.56
Wake-up time (base time; 0 am)		-0.040	-0.061	-0.019	<.01*	<.01*
Sleeping hours		0.004	-0.013	0.020	.66	.66
Television watching	< 1 hour	-0.040	-0.142	0.061	.44	.13
	1 hour -	-0.045	-0.145	0.056	.38	
	2 hour -	-0.050	-0.151	0.051	.34	
	3 hour -	0.004	-0.101	0.110	.94	
	4 hour -	0.000	-0.118	0.119	1.00	
	5 hour -	0.033	-0.136	0.202	.70	
Video game playing	< 1 hour	0.013	-0.012	0.038	.31	.04*
	1 hour -	0.067	0.025	0.109	<.01*	
	2 hour -	0.024	-0.098	0.145	.70	
	3 hour -	-0.062	-0.328	0.205	.65	
Breakfast at age 4 ½ years	Eating irregularly	-0.115	-0.246	0.015	.08	.22
(Base category; eating regularly)	Don’t eat	0.013	-0.143	0.169	.87	
Lunch at age 4 ½ years	Eating irregularly	0.094	-0.115	0.303	.38	.63
(Base category; eating regularly)	Don’t eat	-0.177	-1.077	0.723	.70	
Dinner at age 4 ½ years	Eating irregularly	0.008	-0.081	0.097	.86	.96
(Base category; eating regularly)	Don’t eat	-0.046	-0.462	0.369	.83	
Snack at age 4 ½ years	Eating irregularly	-0.053	-0.092	-0.013	<.01*	.03*
(Base category; eating regularly)	Don’t eat	-0.026	-0.138	0.086	.65	

*Statistically significant at the level of *P* < .05.

^†^BMI: body mass index.

^‡^The threshold parents’ incomes shown in Japanese yen were converted to the US dollar by purchasing power parity rate (1 dollar = 110 yen).

^§^Type 3 effect test is based on an asymptotic chi-square distribution of the likelihood ratio statistic computed from GEE models and it allows one to test for *an effect of interest provided all the other variables are included in the GEE model*.

## Discussion

We studied a large sample of longitudinal data to elucidate trends in obesity in Japanese children aged approximately ≤5 ½ years. Substantial obesity-related factors were gestational period and parental care provider to children. Since the aforementioned cross-sectional studies of Japanese children by Matsushita et al. [12] showed that the prevalence of obesity among countries differs, we adopted different cutoffs from the IOTF and WHO standards for defining obesity and overweight [10,20]. With the adopted criteria, the BMI trends stratified by obesity and by overweight in our study were similar to those in other countries in that AR occurs earlier in obese and overweight children than nonobese and nonoverweight children [20].

Some obese and overweight children may be suitable for intervention based on not only the early AR but also the rapid increase rate of BMI afterward; the results of GEE analysis, which gives us robust parameter estimates, indicated that on average 5 ½ year-old overweight children exhibited 1.74-fold higher annual increment in BMI compared with aged-matched nonoverweight children. Importantly, our survival analysis showed that obese children had approximately 50% higher risk of undergoing AR by age 4 ½ years than nonobese children while the difference in AR prevalence between obese and nonobese children at age 4 ½ years was 9%.

Cole discussed that, instead of using AR, observing concurrent BMI levels and growth trends during childhood allow us to directly predict obesity [31]. However, our survival analysis showed that it is important to detect AR early in Japanese children to intervene to reduce obesity and that AR plays a significant role as an indicator for obesity. A potential physiological reason is given by Campbell et al. who reported that longitudinal changes in body composition explain the timing of AR [32].

Early intervention itself such as counseling parents of high risk children is feasible and practical in Japan if, for example, it becomes a part of the maternal and child health (MCH) handbook program [33]. This system mandates all parents or guardians to communicate doctors and health professionals at any clinics and hospitals using a MCH booklet or *boshi-techo.* The Japanese government also mandates a child’s periodical health check-up from birth until he or she enters kindergarten. The MCH program has been employed as a national program in 11 countries such as Indonesia, Thailand, South Korea and it has been being introduced in 20 countries such as Brazil, Peru, and India. Intervention for children’s health is also feasible in these countries using this program.

This study identified several factors affecting BMI values during 5 ½ years. Children born at <37 weeks of gestation had higher BMI values than those born after longer gestations. This finding does not seem to agree with previous studies that suggested no significant association between gestational period and obesity [34,35]. To reach a definite conclusion, however, further biological and epidemiological studies are required to investigate the relationship between duration of gestational period and mechanism of physical growth. If our finding is further supported, then attention should be paid to a rapid increase in BMI in premature infants.

Regarding parental care providers of infants, those other than parents or grandparents accounted for 3.9% at the 1st follow-up survey (at age 6 months) and 56.3% at the 5th follow-up survey (at age 4 ½ years). The percentage varied widely among the surveillances, suggesting that children were taken care of by their mothers at home during infancy and early childhood, whereas they were mostly taken care of in daycare during daytime when they were aged 4 ½ years. BMI increased more among infants who were looked after by others (nonfamily members) than their parents or grandparents at around age 6 months and during later surveys. This might imply that insufficient attention was being paid to diet and exercise in daycare settings. Another possibility is that nonfamily members cannot provide instructive discipline including dietary guidance to children.

Previous studies have indicated that time spent for watching television is a contributory factor to obesity [36,37]. We found no significant association between BMI increase and time spent for watching television. Even if watching television discourages children’s physical activity, it seems to be hard to detect a relationship between time spent for watching television and BMI increase in Japanese children provided that 98.9% of them watch television. On the contrary, the effect of video game playing on BMI was statistically significant; children who played video games for 1–2 hours daily had a 0.068 higher annual increment of BMI than those who did not play video games. However, the size of this BMI increment does not seem to have a clinically meaningful effect. Playing video games for ≥2 hours was not significant in the same analysis and only 1.4% of children reported playing ≥2 hours.

Among the other candidate explanatory variables, we showed that children who woke up later experienced smaller increases in BMI than those who awoke earlier. However, length of sleep hours was not a significant factor. Similarly, habit of consuming snack was a statistical significant effect especially among children who ate snacks regularly or irregularly, but regular snack consumption merely increased BMI against irregular snack consumption. Given that the study sample was quite large, one possible explanation for the statistical significances with insignificant effect sizes would be the result of type I error (false positive) [38]. Nevertheless, more detailed and effective measurements for sedentary or energy intake behaviors against childhood obesity, in addition to watching television, playing video games, and snack consumption, should be developed and explored in future research.

We acknowledge that our study has several limitations. The 21st Century Longitudinal Survey in Newborns was not necessarily designed to consistently collect the same survey items for our study. This is because the aim of the national survey was to understand multidimensional aspects of life in Japanese children and it is not for specifically evaluating physical development. For example, at the survey of age 6 months, birth weight and birth height were collected but the current weight and height were missing. In addition, weight and height were self-reported by the parents. Although Japanese parents customarily refer to weight and height recorded in the aforementioned booklet, *boshi-techo* provided by the MCH handbook program, which contains reliable medical data verified by their physicians, some recall errors may still have been introduced in the study data. As another instance, because detailed data on food intake of children were not available, we were not able to thoroughly investigate dietary habits. The present study identified a statistically significant relationship between irregular snack consumption and BMI as other studies previously indicated a relationship between diet and obesity [14,39,40]. However, composite data on nutrition was not available which would have been required for further exploring the influence of dietary habit. Lastly, we assumed that our data was *missing completely at random* (*MCAR*) after our careful validity check described in the statistical section. Ideally, our findings could be verified with another large cohort of Japanese children of which data mechanism is *missing at random* (*MAR*). However, to the best of our knowledge there is not such a dataset available.

The data collected by the Japanese government is highly valuable and our findings can be used to improve the health policy in the country. Our study approach and conclusions will be also informative for investigators who study ethnic populations beyond widely studied western countries.

## Conclusion

We studied data from the first longitudinal survey of Japanese children. With a BMI cutoff from the literature that compensates for ethnic differences, AR was observed earlier in obese children than in nonobese children, similar to those reported in Western countries. Furthermore, obese children had approximately 50% higher risk of undergoing AR by age 4 ½ years compared to nonobese children. We found several socio-demographic risk factors affecting level of BMI; children who were born with shorter gestation period and have non-family members as their primary caretakers had higher risk of BMI increase. The sample size of government survey was quite large, which allowed us to have high statistical power and thus good confidence in our results.

## Abbreviations

BMI: Body mass index; AR: Adiposity rebound; GEE: Generalized estimating equations; MCAR: Missing completely at random.

## Competing interests

The authors declare that they have no competing interests.

## Pre-publication history

The pre-publication history for this paper can be accessed here:

http://www.biomedcentral.com/1471-2458/14/334/prepub
